# Influenza A H3N2-Associated Meningoencephalitis in an Older Adult With Viral RNA in Cerebrospinal Fluid: Case Report

**DOI:** 10.3389/fneur.2022.874078

**Published:** 2022-04-25

**Authors:** Yu-chao Dou, Yu-qing Li

**Affiliations:** Tianjin Key Laboratory of Cerebrovascular and of Neurodegenerative Diseases, Tianjin Dementia Institute, Department of Neurology, Tianjin Huanhu Hospital, Tianjin, China

**Keywords:** influenza A H3N2, adult, influenza associated encephalitis, metagenomic next-generation sequencing, cerebrospinal fluid

## Abstract

Influenza-associated encephalopathy (IAE) is most frequently observed in young children, but less reported in adults. Diagnosis of IAE is difficult, as clinical presentations vary significantly and the influenza virus is rarely detected in cerebrospinal fluid (CSF). Herein, we described the case of an older adult presenting with acute meningoencephalitis due to an influenza A (H3N2) infection and the influenza A (H3N2) RNA is detected in cerebrospinal fluid. To the best of our knowledge, this is infrequently reported in the literature. We emphasize that, in adults presenting with acute viral encephalitis, clinicians should consider an influenza infection as part of the differential diagnosis and that metagenomic next-generation sequencing of CSF for IAE may help establish an accurate diagnosis. It must be emphasized that the administration of steroids in a timely manner following the onset of symptoms may yield a better outcome in patients.

## Introduction

Influenza-associated encephalopathy (IAE) is more common in young children than in adults. The most severe category of IAE is acute necrotizing encephalopathy (ANE), first described in Japan in 1995, and is characterized by the sudden onset of fever, convulsions, coma, and even death. Symmetric inflammatory brain lesions are generally noted on neuroimaging (1, 2). Since the global pandemic of novel influenza A H1N1 in 2009, there has been a sustained rise in the number of cases of IAE (3). While influenza A H1N1-associated encephalopathy is reported more than others, the prevalence of H3N2 associated encephalitis has been increasing in recent years (4). However, only a small number of cases have been reported in China. Here, we describe a rare case of a 70 year old male patient presenting with meningoencephalitis due to an influenza A H3N2 infection.

## Case Report

A 70 year old male patient with a history of sinusitis and nasal polyps presented to the emergency department with a 2 day history of headache and without an of prodromal respiratory symptoms in the prior 2 weeks. He had no history of influenza virus vaccination.

**Figure 1:**
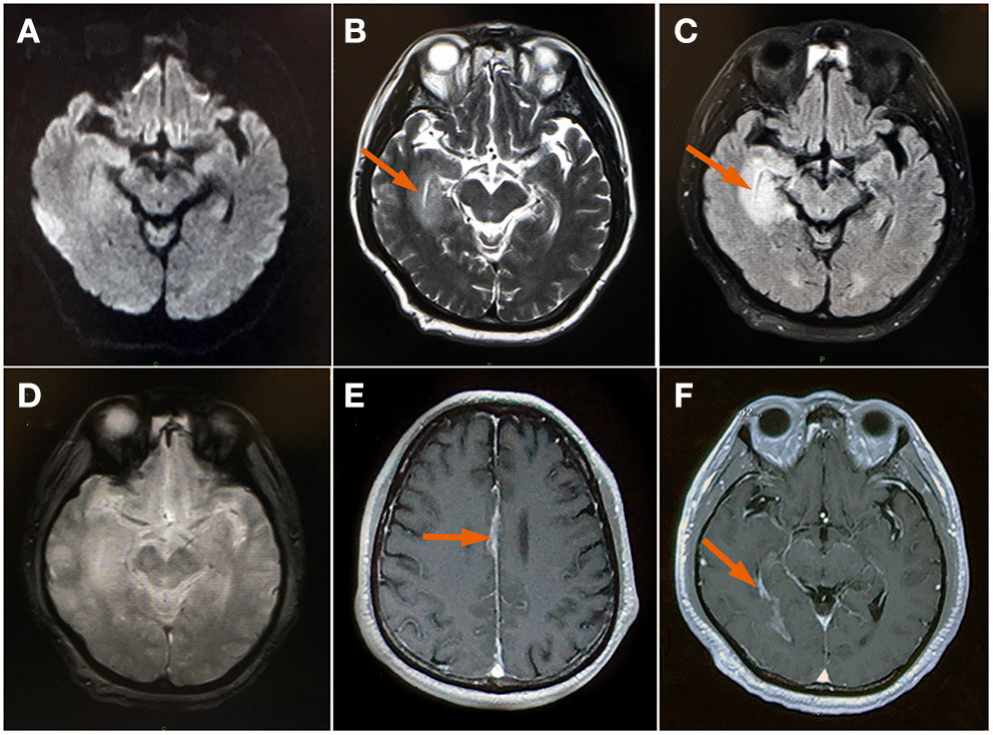
MRI brain **(A)** Axial DWI and **(D)** GRE sequence showed insignificant findings; **(B)** axial T2WI showed bright signal axial (arrow); **(C)** FLAIR image showed an edematous swollen temporal lobe (arrow); **(E,F)** Contrast-enhanced images demonstrated a fine linear enhancement of cerebral falx and bilateral choroid (arrow). DWI, diffusion-weighted imaging; FLAIR, fluid-attenuated inversion-recovery; GRE (gradient echo); T2WI, T2 weighted image.

Upon arrival to the emergency department, the patient presented with severe persistent headache, no vomiting, no disturbance of consciousness and no limb convulsions. Neurological examination revealed clear consciousness, fluent speech, equal pupils, pupil light reflex is positive, full eye movement, no diplopia or nystagmus, bilateral facial symmetry, positive neck stiffness, negative Kerning sign, Brudzinski sign and bilateral Babinski signs. The muscle strength and tension of limbs are normal. His chest CT, routine blood and biochemical analyses showed insignificant findings. Brain magnetic resonance imaging (MRI) revealed right temporal lobe lesions (shown in Figure 1) and he was given treatment with gabapentin for his headache. The gadolinium enhanced brain MRI demonstrated a fine linear enhancement of the bilateral frontal sulcus, cerebral falx, bilateral choroid, and bilateral ventricular ependyma (shown in Figure 1). Broad-spectrum antimicrobial agents, intravenous ceftriaxone and ganciclovir, were initiated for presumed infectious meningoencephalitis.

On the 3rd day of admission to the emergency department, the patient developed a fever up to 39°C and was transferred to the Department of Neurology. There were no disturbing symptoms after hospitalization. Neurological examination revealed clear consciousness, fluent speech, equal pupils, pupil light reflex is positive, full eye movement, no diplopia or nystagmus, bilateral facial symmetry, positive neck stiffness, negative Kerning sign, Brudzinski sign and bilateral Babinski signs. The muscle strength and tension of limbs are normal. Laboratory data showed a normal white blood cell count (8.69 × 10^9^/L), but excessively elevated levels of aspartate aminotransferase (80 U/L) and alanine aminotransferase (157 U/L). A CSF analysis showed elevated total protein levels of 163 mg/dL, elevated total cell count of 720 × 10^6^/L, CSF white blood cell count of 650 × 106/L (42% polymorphonuclear leukocytes, 58% lymphocytes), elevated CSF immunity (IgG = 221 mg/L, IgA = 42.7 mg/L, albumin = 750 mg/L, IgM = 1.16 mg/L), decreased glucose of 2.28 mmol/L, decreased chlorides of 118 mmol/L, and Gram-staining results were negative, with no microbial growth.

Influenza A H3N2 was detected in the CSF from metagenomic next-generation sequencing and the SARS-Co-2 and Herpes simplex was not detected in the CSF, spectrum of autoimmune diseases and Spectrum of demyelinating disorders of the central nervous system test was negative from the CSF. Therefore, a diagnosis of influenza A (H3N2) associated meningoencephalitis was made based on the MRI findings, clinical presentation, and CSF analysis and treatment with oral oseltamivir at 75 mg twice a day was initiated on the second day of admission to the Department of Neurology. Simultaneously, intravenous methylprednisolone pulse therapy (at 80 mg a day for 5 days) was started, followed by a slow tapering of oral methylprednisolone. After a 5-day course of oseltamivir and methylprednisolone, the patient began to gradually recover and was completely recovered by day thirteen. The control examination of the CSF before discharge from the hospital showed total protein levels of 52 mg/dL, total cell count of 40 × 10^6^/L, CSF white blood cell count of 30 × 10^6^/L (13.3% polymorphonuclear leukocytes, 86.7% lymphocytes), CSF immunity (IgG = 62.7 mg/L, IgA = 9.2 mg/L, albumin = 283 mg/L, IgM = 0.88 mg/L), normal glucose at 2.28 mmol/L, and normal chlorides at 123 mmol/L, and Gram-staining results were negative, with no microbial growth. The above results are better than before. He was discharged without any subsequent encephalitic or neuropsychiatric manifestations. He was prescribed a slow tapering of oral methylprednisolone.

**Figure 2:**
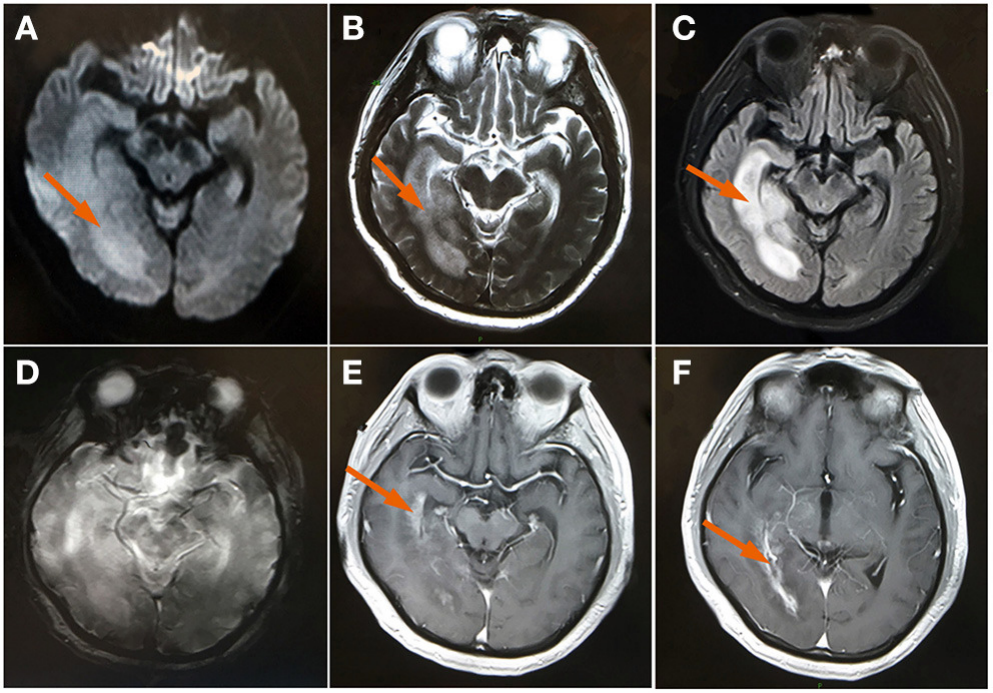
MRI brain **(A)** Axial DWI and **(B)** axial T2WI showed a high signal and the size of the edematous area showed a marked increase at the right temporal occipital lobe (arrow); **(C)** FLAIR image showed a swollen, edematous temporal occipital lobe (arrow); **(D)** axial GRE sequence at the same level with the high signal of T2WI; **(E,F)** A contrast-enhanced image demonstrated an abnormal enhancement of the right temporal occipital lobe and bilateral choroid (arrow). DWI, diffusion-weighted imaging; FLAIR, fluid-attenuated inversion-recovery; GRE, gradient echo image; T2WI, T2 weighted image.

One month later, the patient developed a fever and headache again. Upon inquiry, the patient had stopped methylprednisolone on his own and did not comply with the doctor's order. A brain MRI DWI image and T2-W image demonstrated an abnormal signal in the right temporal occipital lobe, which showed an increase in the range of the lesion (shown in Figure 2). A contrast-enhanced coronal T1-W image demonstrated obvious abnormal enhancement in the right temporal occipital lobe and bilateral choroid (shown in Figure 2).

A lumbar puncture showed an elevated total protein of 1,030 mg/dL, elevated total cell count of 220 × 10^6^/L, white blood cell count of 190 × 10^6^/L (32% polymorphonuclear leukocytes, 68% lymphocytes), elevated CSF immunity (IgG = 113 mg/L, IgA = 17.8 mg/L, albumin = 560 mg/L, IgM = 3.27 mg/L), normal glucose at 2.82 mmol/L, and normal chlorides at 122 mmol/L. Additionally, the Gram-staining results were negative, with no microbial growth. The CSF metagenomic next-generation sequencing and CSF neuronal/inflammatory antibody test were insignificant. Non-specific autoimmune encephalitis caused by viral infection was considered and intravenous methylprednisolone pulse therapy (500 mg daily for 3 days) was started, followed by a slow tapering of oral methylprednisolone. The subject had completely recovered by day nine and he was discharged without any symptoms. He was given instructions to continue the slow tapering of oral methylprednisolone and to follow-up after 3 months so long as he was without any discomfort.

## Discussion

IAE is a serious complication of influenza infection and frequently begins with a prodrome of respiratory symptoms, followed by a disturbance of consciousness and seizures. In young children, altered consciousness tends to be the most frequent neurological manifestation, while respiratory symptoms are also commonly present at admission (5). The interval between the onset of respiratory symptoms and IAE ranges from 1 to 14 days (6). Influenza is rarely detected from the CSF, which may be due to a low viral load or the clearance of the virus from the CSF prior to sampling (7). Since the world pandemic of influenza A (H1N1) in 2009, there have been a few reports of IAE in various countries, which generally focus on the cases with severe neurological complications, with fewer reports of mild cases (8).

Here we describe a rare case of an older adult patient with acute meningoencephalitis associated with an H3N2 influenza infection. Different from previous reports, this case is characterized by an older adult with no prodromal respiratory symptoms or mild neurological complications, and the influenza was detected within the CSF.

On an MRI, the lesions of IAE are typically multifocal bilateral symmetric and primarily involve the thalami, cerebral periventricular white matter, brainstem tegmentum, or pons and cerebellum, and can also be manifested as diffuse cortical involvement and diffuse cerebral edema, however, meningeal enhancement has been rarely reported (9). This patient's neuroimaging findings were different from those in previous reports, in that the lesion was found primarily in the unilateral temporal lobe with no necrosis. Interestingly, the meninges showed significant enhancement, which has only been limitedly reported previously.

Until now, the pathogenesis of IAE has not been clear; previous studies suggested that the lack of a detectable influenza in CSF suggest that IAE is likely due to post-infection inflammation or immune-mediated responses rather than the direct effect of the virus (10). CSF cytology, protein, and glucose levels are normal in most cases, but mild pleocytosis or elevated protein levels are occasionally observed (11).

While previous reports differ, in this patient, influenza was detected in the CSF and a CSF analysis showed pleocytosis, elevated protein levels, and elevated IgG and IgA. This suggests that a nervous system injury may be related to an inflammatory or immune-mediated response and therefore may also be a direct effect of virus.

Currently, there is no recommendation for standard of care for this disease. Treatment is primarily supportive and may involve intensive care. Antiviral therapy with oseltamivir is recommended, particularly in patients who present within 48 h of the onset of symptoms, to reduce viral replication (6). The administration of steroids within 24 h after the onset of symptoms tends to yield a better outcome in those patients without brainstem lesions (12). Given his symptoms, neuroimaging, and laboratory tests, the patient described here received antiviral medication and corticosteroids, which could explain the therapy failure at the beginning in our case. due to a delay in the diagnosis with the subsequent start of steroid therapy and the patient's failure to follow the doctor's order to take oral steroids.

In summary, the findings on the neuroimaging and CSF examination in this case vary from previous reports. A molecular analysis of the CSF supports the hypothesis that adult IAE may be caused by a direct invasion of the brain by the influenza virus and is also the result of the immune response to the virus, but further research is still needed. Furthermore, it must be emphasized that timely steroid treatment in the early stages of the disease is extremely important.

## Data Availability Statement

The raw data supporting the conclusions of this article will be made available by the authors, without undue reservation.

## Ethics Statement

The studies involving human participants were reviewed and approved by the Medical Ethics Committee of Tianjin Huanhu Hospital. The patients/participants provided their written informed consent to participate in this study.

## Author Contributions

Y-cD and Y-qL collected data and drafted and revised articles. Both authors contributed to the article and approved the submitted version.

## Funding

This work was supported by the Tianjin Key Medical Discipline (Specialty) Construction Project.

## Conflict of Interest

The authors declare that the research was conducted in the absence of any commercial or financial relationships that could be construed as a potential conflict of interest.

## Publisher's Note

All claims expressed in this article are solely those of the authors and do not necessarily represent those of their affiliated organizations, or those of the publisher, the editors and the reviewers. Any product that may be evaluated in this article, or claim that may be made by its manufacturer, is not guaranteed or endorsed by the publisher.
